# Evidence based politics: knowing and acting today to shape tomorrow

**DOI:** 10.1186/1753-6561-7-S5-O13

**Published:** 2013-08-30

**Authors:** Ignazio Cassis

**Affiliations:** 1 National Council, Switzerland

## 

The role of scientific evidence in influencing political decisions that affect the future and the importance of research in generating such evidence is highlighted in the context of the Swiss federation. Within the Swiss federation the responsibilities are balanced between the confederation and the cantons. Currently Switzerland faces several challenges such as epidemiological transition, increase in chronic non-communicable diseases, increasing social costs, increasing health care costs, need for more medical professionals and health inequalities. Therefore we need more scientific knowledge to tackle these problems. Considerable investment in research infrastructure is needed in the form of cohorts and biobanks. Two new federal laws have been enacted which pertain to prevention, health promotion, managed care and disease management. However such laws need scientific support and collaboration across political, economic, academic and social forces.

